# Commonly Used Types and Recent Development of Ankle-Foot Orthosis: A Narrative Review

**DOI:** 10.3390/healthcare9081046

**Published:** 2021-08-13

**Authors:** Yoo Jin Choo, Min Cheol Chang

**Affiliations:** Department of Rehabilitation Medicine, College of Medicine, Yeungnam University, Daegu 42415, Korea; cyj361@hanmail.net

**Keywords:** ankle-foot orthosis, orthosis, review

## Abstract

(1) Background: ankle-foot orthosis (AFO) is the most commonly prescribed orthosis to patients with foot drop, and ankle and foot problems. In this study, we aimed to review the commonly used types of AFO and introduce the recent development of AFO. (2) Methods: narrative review. (3) Results: AFO prevents the foot from being dragged, provides a clearance between the foot and the ground in the swinging phase of gait, and maintains a stable posture by allowing heel contact with the ground during the stance phase. In clinical practice, the most commonly used AFO include plastic AFO, walking boot, UD-Flex, and carbon fiber AFO. In addition, for compensating the demerits of these conventional AFOs, new types of AFOs, including AF Servo, TurboMed, three-dimensionally printed AFO, and AFO made from kenaf composites, were developed. (4) Conclusions: we think that our review can guide clinicians in selecting and prescribing the appropriate AFO for each patient in accordance with their specific physical conditions.

## 1. Introduction

Ankle-foot orthosis (AFO) is a commonly used orthosis in patients having weakness in the ankle dorsiflexor or plantarflexor muscles due to several disorders such as stroke, cerebral palsy, spinal cord injury, and peripheral nerve injury. In addition, it can be used in patients with arthritis or ankle and foot deformities or fractures [1,2,3,4,5,6]. AFO assists walking by maintaining the alignment of and stabilizing the ankle and foot, and prevents and corrects ankle and foot deformities [7,8]. AFO prevents the foot from being dragged, provides a clearance between the foot, and the ground in the swinging phase of gait, and maintains a stable posture by allowing heel contact with the ground during the stance phase [9,10].

In this study, we aimed to review the commonly used conventional types of AFOs and introduce the recently developed AFOs. In addition, we aimed to investigate and compare the advantages and disadvantages of conventional and recently developed AFOs and provide useful basic data for prescribing AFOs in clinical practice.

## 2. Conventional AFO Used in Clinical Practice

### 2.1. Typical Plastic AFO

**Figure 1:**
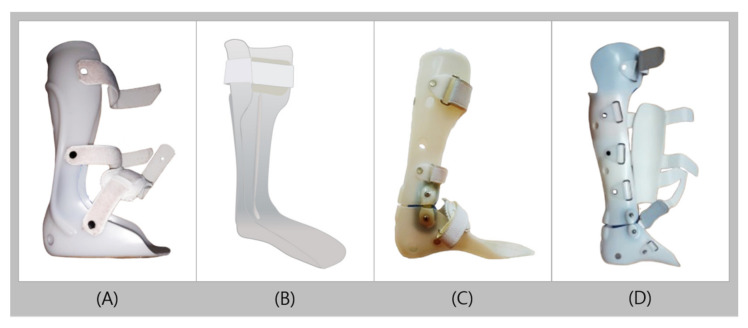
Types of plastic ankle-foot orthoses (**A**): solid ankle-foot orthosis, (**B**): posterior leaf spring orthosis, (**C**): hinged ankle-foot orthosis, and (**D**): patellar tendon-bearing ankle-foot orthosis.

Plastic AFO (PAFO) is mainly made of thermoplastics such as polypropylene and is one of the most widely used orthosis in clinical practice owing to its numerous advantages such as its relatively low cost, good aesthetics, being easy to clean, and easy desorption [11,12]. PAFO is customized to the patient’s body, as it is generally produced by casting the patient’s lower extremity to make a positive plaster model. By placing thermoformed plastic to cover the positive plaster model, it produces the orthosis in the exact shape of the model. PAFO commonly consists of a shank shell, foot plate, and Velcro strap, with hinges on ankle joints as needed [13,14]. PAFO can be classified according to the presence of hinges, mainly as solid ankle types without hinges and hinged ankle types with additional hinges. If the purpose is to solely keep the ankle in a neutral position, a solid ankle type is applied, and the trimline is placed in front of the ankle bone to control the medial and lateral stabilities of the ankle (Figure 1A) [15].

Solid AFO (SAFO) is predominantly applied to completely limit the ankle joint movement in patients with foot drop, weak dorsiflexion and/or plantarflexion, ligament injury about the ankle, mild knee instability, and valgus/varus [16,17]. Posterior leaf spring orthosis (PLSO) is a SAFO, but unlike the conventional SAFO, PLSO has a characteristic trimline located behind the ankle and has a leaf-shaped corrugation near the ankle (Figure 1B). The leaf-like creases are intended to strengthen the part of the ankle with the most amount of movement and repeated loadings. The creases act as a spring in the ankle that allows slight dorsiflexion in the mid and terminal stances, and this elasticity can also marginally assist the push-off function in the terminal stance. PLSO is used in the presence of motor weakness in the ankle dorsiflexor caused by conditions such as cerebral palsy and stroke. Owing to its greater elasticity and flexibility than those of regular SAFO, PLSO is suitable for patients with mild cramps or who are more active and have better balance than those for whom SAFO is used [18,19,20]. In addition, as the ankle trimline extends further to the front of the ankle joint, the effectiveness in controlling the instability of the ankle increases. However, the PLSO does not contribute significantly to ankle stability, as the trimline is behind the ankle. Therefore, PLSO has a limitation in controlling valgus/varus [20,21].

**Figure 2:**
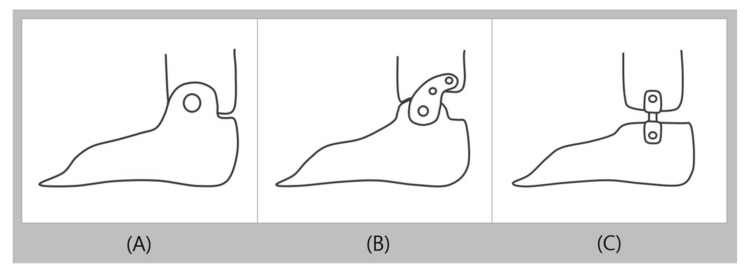
Commonly used types of plastic ankle foot joints (**A**): overlap joint, (**B**): Oklahoma joint, and (**C**): Gillette joint.

The hinged AFO (HAFO) is used when ankle movement is permitted but movement restrictions to a certain extent is required. HAFO is produced by using hinges to connect two pieces, the shank and foot shells, and the hinges are commonly located on the malleolus side (Figure 1C). The hinge on the HAFO allows a certain degree of dorsiflexion that makes it easier for users to walk on uneven surfaces or to climb stairs. It also increases ankle dorsiflexion in the terminal stance and increases ankle plantar flexion during the pre-swing phase, which helps users walk naturally [22]. The commonly used hinged types of PAFO include the overlap, Oklahoma, and Gillette joints (Figure 2). The overlap joint limits plantarflexion by overlapping the shank and foot shells, and fixing it in with a rivet. The Oklahoma joint connects a separate shank shell with the foot shell, which creates a space between the shank shell and the back of the foot shell to allow plantarflexion until the two pieces meet. The plantarflexion can also be completely limited by fitting the shells at 90° without space in between. The Gillette joint, like the Oklahoma joint, connects a separate shank shell with the foot shell, allowing both plantarflexion and dorsiflexion. HAFO is widely used in children with spastic diplegia and patients with spastic hemiplegia after stroke, as it can stretch the ankle plantar flexor to reduce stiffness and reduce disorganized muscle-response patterns. It is also used in the presence of low muscle tone (hypotonia), high muscle tone (hypertonia), flexible pronation or supination, sagittal and/or frontal plane weakness, excessive plantarflexion, and genu recurvatum. However, it should be applied into patients with sufficient control of their knee joints and should not be used for patients with severe mediolateral instability of the ankle [22,23,24].

The patellar tendon bearing AFO (PTB-AFO), unlike other PAFOs, has an additional anterior shell to support weight with the patellar tendon, which helps to reduce the weight load on the heel, ankle, and sole, and therefore reduces pain in each of the mentioned areas (Figure 1D) [25,26]. This is used in cases that require the pressure on the foot to be minimized, such as ulcers, calcanectomy, plantar skin graft, severe foot/ankle trauma, and fractures.

In 2007, Balaban et al. [1] measured the walking parameters and angle of the ankle during walking in 11 children with hemiplegic cerebral palsy according to the presence or absence of a hinged AFO made with a plantarflexion stop at 0° with no dorsiflexion stop. As a result, with a hinged AFO and bare feet, respectively, the mean velocities were 0.89 and 0.70 m/s; single support averages, 0.38 and 0.36 s; double support averages, 0.18 and 0.23 s; and stride lengths, 0.90 and 0.73 m, which demonstrated significant differences in all four components. Furthermore, the angle of ankle dorsiflexion at initial contact was 4.79° with a hinged AFO and −4.28° with bare feet, and the ankle dorsiflexion at mid stance averaged at 11.59° and 5.81°, respectively, which also showed significant differences in the two components. With such results, walking function has been confirmed to improve when hinged AFO is used, as compared with walking barefoot. Abe et al. [27] in 2009 evaluated walking function using an 8-m walk test and functional ambulation category (FAC) with or without using a plastic AFO in 16 hemiplegic stroke patients. As a result, in the patients who used and did not use orthoses, respectively, the stride lengths were 65.7 ± 13.6 and 56.9 ± 13.6 cm; affected-side step lengths, 34.0 ± 10.0 and 30.4 ± 9.4 cm; step widths, 29.8 ± 4.4 and 28.2 ± 5.0 cm; velocities, 22.9 ± 6.8 and 18.1 ± 8.1 m/min; and cadences, 73.3 ± 15.8 and 66.8 ± 21.0 step/min, which all demonstrated significant differences. The FAC score was 3 points in 9 patients (56.3%), 4 points in 7 patients (43.8%), and 5 points in none (0%) of the patients prior to wearing the orthosis. However, after using the orthosis, the FAC score was 3 points in 1 patient (6.3%), 4 points in 5 patients (31.3%), and 5 points in 10 patients (62.5%), which reported significant improvement in walking ability.

### 2.2. Walking Boot (Controlled Ankle Movement Walker, Aircast)

**Figure 3:**
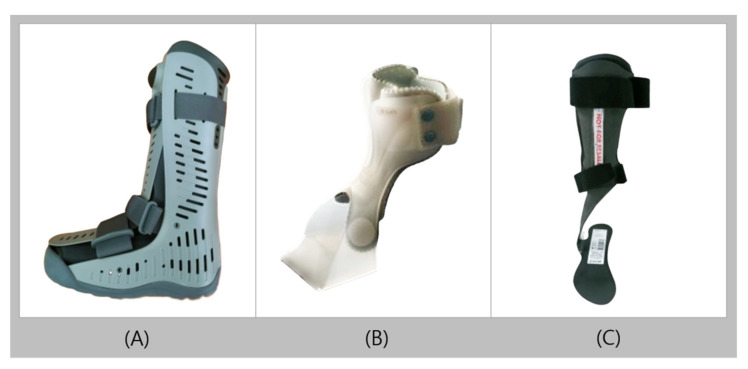
Conventional ankle-foot orthoses (**A**): walking boot, (**B**): UD-Flex, and (**C**): carbon fiber ankle-foot orthosis.

The walking boot (WB) is an orthosis that allows total contact with the anterior and posterior parts of the calf, ankle, and entire foot (Figure 3A). The WB is equipped with inflatable pneumatic blades to maintain stable surface contact between the orthosis and the user’s skin. The pneumatic blades can also reduce edema and shear forces, and separately inflate the particular areas that require inflation for total contact [28]. The entire inner part of the orthosis contains liners to provide cushion for the inner surface. The WB, like a cast, reduces movement by fixing the lower extremities and ankles at 90°, but the front plate of the orthosis can be easily removed to identify and evaluate wounds [28,29,30]. As the bottom surface is commonly produced with a rocker bottom, a more natural and comfortable movement is possible during the toe-off of the initial swing [31]. An angle adjuster can be added when necessary to adjust the orthosis and allow for ankle movement within the required range. The WB is used for acute injuries such as ligament sprains/tears, postoperative stabilization or support, ulcers, or cases with fractures [29,32,33,34,35].

Amaha et al. [36] in 2016 retrospectively evaluated patients who had surgeries for unstable ankle fractures. Of the 47 patients who received follow-up observations for at least 6 months, 25 wore a plaster cast (PC) and 22 wore a WB, and recovery rates were monitored in the two groups. As a result, the time taken for the patient to recover the ability to stand unipedal on the affected side after allowing full weight bearing showed a significant difference, with a mean duration of 3.1 weeks in the PC group and 1.4 weeks in the WB group. The time taken for the patient to recover the ability to walk without crutches was also significantly different, with a mean duration of 4.5 weeks in the PC group and 2.6 weeks in the WB group. This signifies that the WB group demonstrated an outstanding degree of recovery.

### 2.3. UD-Flex

Unlike the conventional AFO, UD-Flex is an orthosis designed to be worn at the front of the foot, with a completely open heel (Figure 3B). The front shell of the orthosis is U-shaped and has flexibility that allows users to bend the ankle sufficiently. In addition, the contact area with the foot and orthosis is small, and the open heel area allows users to receive ground reaction feedback when walking. Therefore, users can actively use their proprioceptive sensibility. they can walk while accurately recognizing their walking pattern, which leads to an even more natural way of walking [28,37]. Users were required to wear shoes one size larger than the normal size for their feet, as the heel was not opened for the existing PAFOs. Comparatively, UD-Flex provides a better solution for this issue and allowed users to wear shoes of the same size for both the affected and normal feet. UD-Flex can be made in sizes according to the length of the foot or can be customized to fit the body of the individual. UD-Flex features a consistent state of 5° dorsiflexion, is ultralightweight, and is easy to wear with one hand, which is advantageous for hemiplegic patients. UD-Flex is applied in patients with foot drop due to mild foot deformity, stroke, and spinal nerve injury, and is also used to relieve pain and prevent foot deformations after tendon reconstruction operation. In addition, UD-Flex is used to improve the walking ability of patients after a certain degree of improvement in symptoms of muscle weakness is attained, rather than in the early stages of the disease.

Bae et al. [37] in 2009 evaluated the function of walking with and without the use of UD-Flex in 12 hemiplegic stroke patients. The results showed a significant difference in the peak ankle dorsiflexion in the swing phase between the two groups, with an ankle dorsiflexion angle of 3.14° ± 6.77° in the UD-Flex group and 0.56° ± 6.40° in the non-UD-Flex group. Such results signify that UD-Flex assists in dorsiflexion during the swing phase of walking and therefore has an enabling effect on natural gait.

### 2.4. Carbon Fiber AFO

Carbon fiber is a material with high stiffness, high tensile strength, resistance to high temperatures, and low weight. Owing to the nature of its material, carbon fiber AFO (CFAFO) is considered to be better than plastic AFO in terms of energy storage capacity, light weight, and durability, but it is not commonly used because of its high cost [38,39,40]. Among the types of plastic orthosis, the solid ankle AFO or posterior leaf spring has a design similar to that of CFAFO, except that in CFAFO, the heel is open and the shell is thin throughout the entire orthosis to reduce the pressure exerted on the user (Figure 3C) [40,41]. In addition, the overall satisfaction of users is high, as the appearance is more modern and the function is superior to the existing orthoses [39,40,42]. The CFAFO, compared with plastic orthosis, enhances the plantarflexor ankle joint moment and energy efficiency, and can improve walking ability, as it increases plantarflexor muscle power [38,43]. CFAFO is used for foot drop, limb proprioception deficiency, M-L instability, mild knee instability, Charcot–Marie–Tooth disease, and poliomyelitis, where no spasticity is evident [39,44,45].

In 2006, Desloovere et al. [46] compared the differences in ankle movements during walking using a CFAFO and without using a CFAFO (barefoot) in 15 children with hemiplegia. As a result, the angle at initial contact, range of motion during push-off, timing of maximum dorsiflexion in stance, angle at mid-swing, mean foot progression angle in stance, and angular velocity at toe-off significantly improved when a CFAFO was worn, as compared with walking barefoot. This result signifies that the ankle range of motion improved when the participants walked using a CFAFO as compared with walking barefoot. In 2009, Moriello et al. [47] applied carbon fiber orthoses in adolescent male patients who had traumatic brain injuries and conducted track-and-field training with exercise reinforcement once a week for 6 weeks, weight support once a week for 15 weeks, and muscle strength training once a week for 17 weeks to confirm the degree of recovery of running ability. In patients who initially had a significant left lower extremity weakness, impaired standing balance, limited endurance, and running limitations of being able to run 10 m by using plastic AFO under the supervision of the coach, after the intervention, muscle strength in the lower extremities increased, the distance ran independently increased up to 1 mile, the propulsion to absorb the force applied to the impact during the absorption phase improved, and the lower extremity extension during the propulsion phase increased.

## 3. Recent Trends of AFO

### 3.1. AF Servo

**Figure 4:**
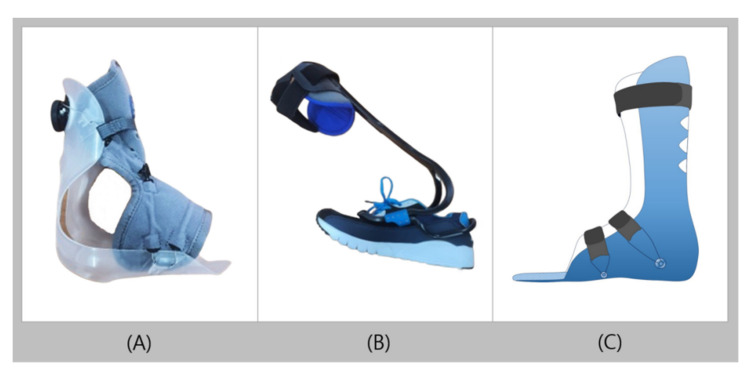
Recent trends of ankle-foot orthoses (**A**): AF Servo, (**B**): TurboMed, and (**C**): three-dimensionally printed AFO.

AF Servo was first introduced in Europe in 2014 and is an orthosis with favorable reviews from many clinicians [48]. AF Servo is made of fabric at the front and plastic at the back, with the trimline located behind the lateral malleolus (Figure 4A). Unlike other customized orthosis, AF Servo is produced ready-made in different sizes and can be worn immediately by operating a dial, which shortens the production period. The BOA fit system, a closing method that enables simple and intuitive fitting, allows the user to easily adjust for an optimal fit by simply operating a dial. As it adheres comfortably according to the body shape of the individual, users can easily fit their feet into shoes. Furthermore, as it does not permit loosening, secondary damage can also be prevented [48]. AF Servo is for patients with mild foot drop; therefore, it is not suitable for patients with severe foot drop who are unable to raise their feet manually. AF Servo is suitable for patients with mild foot drop due to Charcot–Marie–Tooth disease, stroke, Guillain–Barre syndrome, multiple sclerosis, cerebral palsy, and motor neuropathy. Despite its use in clinical practice, to the best of our knowledge, no studies have investigated its effectiveness. In the future, studies evaluating merits of AF Servo are needed.

### 3.2. TurboMed

TurboMed is a dynamic AFO that can be attached to the exterior of various types of shoes, such as sneakers, or sandals, or shoes [49]. Through the mechanical design of TurboMed, the lost dorsiflexion power can be regained, as TurboMed automatically restores the elastic energy according to the gait during walking. TurboMed has an exoskeleton design that can be easily removed from most ready-made shoes without contact with the foot or ankle skin; therefore, users do not have to be concerned about pressure on bone protrusions or wounds (Figure 4B) [50]. In addition, it has no distinction between left and right, and is not largely restricted by shoe sizes (available for shoe sizes 160–340 cm) [50]. It is made of a highly durable plastic material and can be thermoformed to fit the user’s foot width or drooping degree, and its decent adaptability to uneven or inclined ground enables sports activities such as climbing. TurboMed may be applied for patients with weakened dorsiflexor, foot drop, hemiplegia, or peroneal nerve palsy caused by stroke, cerebral palsy, and multiple sclerosis. Other options can be added as needed. For knee hyperextension, calf atrophy, foot slap, and spasticity, an extension stopper can be added to limit the bending of the heel support and reduce the range of motion of the TurboMed body to induce natural walking. If foot inversion is evident, ankle fixation straps can be added to fix the posture [50]. However, TurboMed is less frequently used because of concerns that plastic materials might not be able to withstand the applied load [51]. To date, the number of studies describing the effects of TurboMed are limited. Further studies should be actively conducted to confirm the improvement of walking function and correction of body alignment, or to identify improvements of physical damage such as wounds and edema.

In 2018, Ladlow et al. [52] studied the effect of a passive dynamic AFO with TurboMed-like characteristics on 23 patients with severe lower extremity trauma. After a mean AFO use period of 34 months, 21% and 53% increases in the number of patients able to walk and run independently, respectively, were observed. The proportion of patients reporting no pain also increased from 13% to 31%.

### 3.3. D Printed AFO

The 3D printed AFO is a custom orthosis made by additive manufacturing, which is a method of manufacturing a solid structure by stacking materials in layers (Figure 4C). The AFO, which is produced by 3D printers, can use software to make fine adjustments to bone protrusions or wounds, thereby making it possible to produce orthoses that meet the needs of patients, which is almost impossible to materialize using traditional processes [53]. AFO can be made in various shapes or neatly without leaving surrounding cracks when producing a specific area to be thick or thin or creating perforations for breathability [54,55]. The advantages of 3D printed AFO include rapid production when most conventional orthoses require a considerable amount of time to manufacture because the individual parts must be combined manually; reproducing with the same quality at any time, as the pieces can be easily duplicated; the freedom of users to choose colors; and the relatively low price [56,57,58]. In addition, the light weight eliminates the considerable strain of wearing the orthosis, and the quality water resistance makes it easy to clean. The material has excellent durability, as it is made of a nylon-based polymer with a high level of stiffness and impact strength, or thermoplastic polyurethane, which is one of the non-toxic and highly flexible thermoplastic filaments [56,57]. The 3D printed AFO can be applied in patients with conditions such as foot drop, Charcot–Marie–Tooth disease, and plantar fasciitis caused by conditions such as stroke, cerebral palsy, and multiple sclerosis [56,58].

In 2019, Xu et al. [58] randomly divided 60 patients with plantar fasciitis into a group wearing prefabricated AFO and a group wearing customized 3D printed AFO, and compared the degree of pain after 8 weeks. As a result, the mean visual analog scale score decreased from 8.72 ± 3.93 to 5.25 ± 1.22 in the group wearing prefabricated AFO and from 7.34 ± 3.43 to 3.12 ± 0.51 in the group wearing 3D printed AFO, demonstrating significantly greater pain control effects than the group that did not use prefabricated AFO.

### 3.4. Kenaf Composites

**Figure 5:**
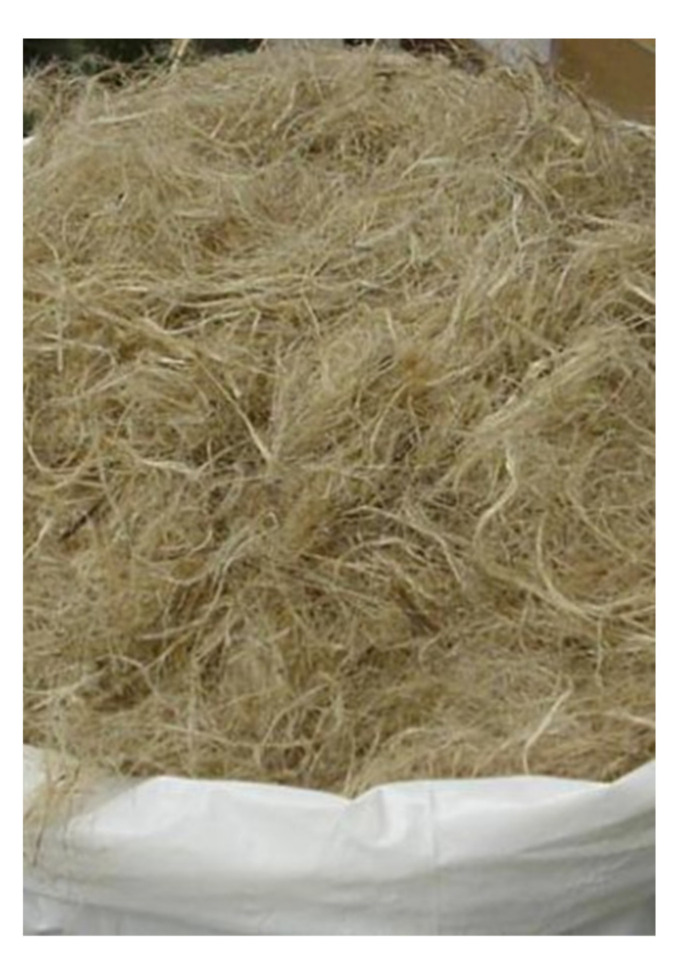
Kenaf composites.

Kenaf composite (Figure 5) is made of natural fibers with strong durability that meets the minimum criteria for mechanical properties required for AFOs [59]. Kenaf is a woody base plant that grows up to 5.5 m in 6–8 months; therefore, when used as a composite, its availability and production costs are not a problem [59,60]. In addition, Kenaf fibers have a trait of absorbing oils and liquids, which can help reduce the possibility of skin irritation, as it can absorb sweat when used as an orthosis [59,61]. However, as kenaf alone is insufficient to withstand the minimum strength applied to the orthosis, it is better to combine the reinforcement material (fiber) with the matrix (resin) to improve the durability of the orthosis [59,60,61]. When producing AFO using Kenaf composites, it is possible to maintain the strength and stiffness of the material while reducing cost and weight, and AFO that are eco-friendly and recyclable can be produced [59]. In 2019, Shahar et al. [59] discovered that carbon fiber composites are the best material for AFOs owing to their low weight and high strength when comparing with the existing materials such as wood, metal and leather, plastic, and carbon fiber composites. However, by mentioning that the cost of using plastic orthosis is higher owing to the high cost of raw materials, Shahar et al. stated that Kenaf composites can be used as a replacement for carbon fiber composites or plastics and for additive manufacturing using 3D printing technology; therefore, the use of Kenaf composites is expected to increase in the future. However, as no studies have been conducted on the effect of AFO made of Kenaf composites, such studies must be conducted.

## 4. Conclusions

In this study, we described the most commonly used and recently developed AFO. Compared to conventional AFOs, recently developed AFOs have better durability, shorter production time, more sophisticated shape-making ability, easier donning, or improved appearance, depending on their types. However, these superior qualities do not necessarily preclude conventional AFOs. The convergence of high-intensity materials and high technology may be economically burdensome, and patients may not want to prematurely use recently developed AFOs because they have no confidence in the products. A variety of new AFOs have been developed, but the most widely used in clinical practice is plastic AFOs because they are relatively inexpensive, vary in type depending on the additional materials used, and can be worn regardless of shoe type. However, plastic AFOs are slightly less durable than recently developed AFOs.

The selection of an appropriate AFO that considers both the physical and psychological state of the user is important to achieve the most successful rehabilitation and increase convenience in daily living. Because different AFOs have different indications, contraindications, features, and user preferences, the appropriate AFO should be selected depending on the status of the user. Future research should consistently be conducted to continuously update AFO selection guidelines and systematically classify which AFO type is most effective for each disease, increasing user preference. In addition, the effectiveness of AFOs that are still being developed should be obtained through clinical trials.

We consider our review to be useful for clinicians when prescribing the appropriate AFO for the specific needs of patients in the future. In addition, this review provides information to broaden the choices of AFO during AFO prescription by clinicians.

## Data Availability

No new data were created or analyzed in this study. Data sharing is not applicable to this article.
